# Supplementary material for: Stereotyped Subclones Revealed by High‐Density Single‐Cell Lineage Tracing Support Robust Development
Source: Adv Sci (Weinh). 2025 Apr 30;12(30):2406208. doi: 10.1002/advs.202406208 (PMC12376543; doi:10.1002/advs.202406208)
Supplement: Supplementary file 1 — Supporting Information [file ADVS-12-2406208-s002.pdf]

ADVANCED SCIENCE
Open Access

Supporting Information

for *Adv. Sci.*, DOI 10.1002/advs.202406208

Stereotyped Subclones Revealed by High-Density Single-Cell Lineage Tracing Support Robust Development

*Xiaoyu Zhang, Zizhang Li, Jingyu Chen, Wenjing Yang, Xingxing He, Peng Wu, Feng Chen, Ziwei Zhou, Chenze Ren, Yuyan Shan, Xiewen Wen, Vassily A. Lyubetsky, Leonid Yu. Rusin, Xiaoshu Chen and Jian-Rong Yang**

Stereotyped Subclones Revealed by High Density Single-Cell Lineage Tracing Support Robust Development

Xiaoyu Zhang, Zizhang Li, Jingyu Chen, Wenjing Yang, Xingxing He, Peng Wu, Feng Chen, Ziwei Zhou, Chenze Ren, Yuyan Shan, Xiewen Wen, Vassily A. Lyubetsky, Leonid Yu. Rusin, Xiaoshu Chen, Jian-Rong Yang,

Supporting Information

Supplementary Text. Detailed computational procedure of mDELTA

Video S1. A typical alveolosphere formed by the directed differentiation procedure

(video in a separated file)

Figure S1. Reliability of the directed differentiation and experimental lineage tracing

Figure S2. Quality of the directed differentiation

Figure S3. Quality of the lineage tracing

Figure S4. More details on the editing status of the lineage barcode

Figure S5. Transcriptional divergence among sub-CLTs

Figure S6. Transcriptional memory in individual cell types

Figure S7. Heritability of descendent cell type compositions and single-cell transcriptomes

(Supplemental tables below are provided in a separated file)

Table S1. Designed lineage barcode sites and sgRNAs

Table S2. Summary statistics of single-cell transcriptomes

Table S3. Number of passes required *versus* sequencing quality of PacBio HiFi-reads

Table S4. List of unique (cell barcode and UMI) lineage barcode alleles and their editing events

Table S5. List of the representative lineage barcode of each cell by their editing events

Table S6. Structure of the constructed cell lineage trees

Table S7. List of analyzed GO terms enriched with stage-specific DEGs

Table S8. List of primers used

Supplementary Text. Detailed computational procedure of mDELTA

mDELTA algorithm to find homeomorphic sub-CLTs

We have previously developed a tree alignment algorithm known as the *D*evelopmental C*e*ll *L*ineage *T*ree *A*lignment (DELTA) algorithm[1]. Briefly, DELTA compares two cell lineage trees (CLTs) and determines the optimal alignment (see below) between them. DELTA has been shown to be capable of identifying sub-CLTs that have highly similar developmental programs (e.g., bilaterally symmetric CLT pairs or CLTs displaying highly similar expression trajectories of key regulatory genes) as well as homeotic cell fate transformations resulting from genetic manipulations[1]. However, DELTA is only applicable to binary (all internal nodes are bifurcating) CLTs with qualitatively labelled terminal nodes (neurons, muscles, etc). To analyze CLT data obtained in this study, we have developed mDELTA, a modified DELTA algorithm that can handle CLTs with multifurcating internal nodes and quantitatively labeled (i.e. with single-cell transcriptomes) terminal cells. Below are the algorithmic details of mDELTA.

Definition of tree and sub-tree in the content of cell lineage trees

A cell lineage tree (CLT), as obtained by our genomic-barcoding-based experiment, can be described as a directed, acyclic, connected graph $T = (V, E)$, where V and E are collections of all nodes (*v*ectors) and *e*dges in the tree, respectively. Each node $v \in V$ represents a single cell, and each edge $e \in E$ represents a descendant relationship from a mother cell to its daughter (or granddaughter, or grand-granddaughter, etc) cell. The number of edges attached to a node (regardless of their direction) is called the degree of the node, referred to as d_v. A terminal node of the CLT has $d_v = 1$, which represents a terminal cell recorded by the CLT, although it is not necessarily the terminal of development (i.e., further divisions of the "terminal" cells are just not recorded as part of the CLT). Furthermore, each terminal node is associated with a single-cell trascriptome that reflects the cell fate/type/state. The nodes with $d_v \geq 2$ are internal nodes representing ancestor cells that have undergone further division recorded by the CLT. The root of the CLT is one special internal node without any incoming edges (i.e., no ancestor cell older than this node is recorded by the CLT). Note that unlike CLTs commonly discussed in nematodes such as *C. elegans*, we ignored the length of each edge (i.e. the temporal duration of the cell cycle) and the order of nodes sharing an immediate mother node, because these information is not available from genomic-barcoding-based lineage-tracing experiments. In this study, the mDELTA algorithm is designed to handle CLTs with internal nodes that have $d_v > 3$, whereas our previously developed DELTA algorithm can only handle CLTs with roots having $d_v = 2$ and all other internal nodes having $d_v = 3$.

A subtree T_v^u is a connected subgraph of a tree $T = (V, E)$. T_v^u contains all nodes $v' \in V$ such that the path between u and v' starts with the edge $(u \rightarrow v)$, as well as all the edges attached to these nodes except $(u \rightarrow v)$. The root of the subtree T_v^u is therefore *v*.

The overall algorithmic objective of CLT alignment

Given a query tree $Q = (V, E)$ and a subject tree $S = (V', E')$, an isomorphic alignment is a bijection $A: V \leftrightarrow V'$, such that for every pair of nodes with $v, u \in V$, we have $(v, u) \in E \Leftrightarrow (A(v), A(\mathrm{u})) \in E'$. Based on two types of biologically informed tree editing operations, namely pruning and merging (defined in the section "Tree editing operations and their costs" below), a homeomorphic subtree alignment *A* between *Q* and *S* is defined as an isomorphic alignment between Q' and S'', where Q' is the result of zero or more pruning and merging in *Q*, and S' is the result of zero or more prunin and merging in *S*. Here all the pruning in *Q* and *S* are collectively denoted as $\pi(A)$, and all merging in Q and S are collectively denoted as $\mu(A)$. If we further denote the alignment score between two nodes (reflecting the similarity in cell state. See the "Cell-to-cell alignment scores" section below) $v \in V$ and $v' \in V'$ as $a(v, v')$, the cost for pruning a subtree $\hat{T}$ as $p(\hat{T})$, the cost for merging an internal node $\hat{v}$ with its mother node as $m(\hat{v})$. The score of a homeomorphic subtree alignment *A* between *Q* and *S* can then be expressed as

$$w(Q, S, A) = \sum_{(v,v') \in A} a(v, v') - \sum_{\hat{T} \in \pi(A)} p(\hat{T}) - \sum_{\hat{v} \in \mu(A)} m(\hat{v}) \quad [1]$$

The objective of mDELTA is to find the optimal *A* (with optimal/highest possible *w*) given *Q*, *S*, *a*, *p* and *m*. Dynamic programming is employed by the mDELTA algorithm to accomplish this. Statistical significance (*P* value) of any alignment between two sub-CLTs was estimated by 1,000 pairs of simulated CLTs created by permutating the terminal nodes of the real CLTs.

The Dynamic Programming procedure of mDELTA

Our mDELTA algorithm utilizes dynamic programming (DP) to find the optimal alignment between two CLTs (*Q* and *S*) by recursively identifying the optimal alignment between their subtrees. It starts from constructing a DP matrix with N_Q row and N_S column, where N_Q and N_S are the total number of nodes in *Q* and *S*, respectively. Each cell of the matrix will store the optimal *w* between the nodes (or the subtree rooted at the node if it is an internal node) represented by the row and the column. For simplicity, we will hereinafter refer to optimal *w* between CLT *Q* and *S* as *w*(*Q*,*S*), with the optimal homeomorphic subtree alignment *A* implicitly indicated.

To fill up the matrix, the *w* between terminal nodes (leaves/tips) are first directly determined by a score that reflects their similarity in cell state (see the "Cell-to-cell alignment scores" section below).

To find the optimal *w* between one terminal node *v* and one internal node *u* with two or more daughter nodes (which could be internal nodes or leaves), all but one daughter nodes (and therefore the subtrees rooted at them) of *u* need to be pruned. For example, in the case of *u* having two daughter cells, we will have $w(v,u) = \max\big(w(v,l) - p(T_r^u),\ w(v,r) - p(T_l^u)\big)$, where $w(v,r) - p(T_l^u)$ is the score given by pruning the left daughter (*l*) of *u*, and $w(v,l) - p(T_r^u)$ is the score given by pruning the right daughter (*r*) of *u*. The optimal choice (indicating how should *v* and *u* be aligned) was stored for later traceback.

To find the optimal *w* between two internal nodes *v* and *u*, both with multiple daughter nodes, mDELTA uses the Hungarian algorithm (also known as the Kuhn–Munkres algorithm). Let us denote the daughter nodes of *v* and *u* respectively as D_v and D_u $(|D_v| = d_v - 1,\ \ |D_u| = d_u - 1)$. When the scores between any pair of daughter nodes are known (already calculated as smaller subproblems in the dynamic programming procedure), the Hungarian algorithm finds the optimal (with maximum total score) sets of pairs each with one node from D_v and one node from D_u. The Hungarian algorithm results in each node from D_v being paired with zero or one node from D_u, and each node from D_u being paired with zero or one node from D_v. Let us denote the total scores of all node pairs found by the Hungarian algorithm as *H*(v,u), and all nodes that have paired with no one and need to be pruned as $\pi(v,u)$. Furthermore, there is another biologically meaningful way of finding the optimal *w* between *v* and *u* that involves merging one or more of their daughter nodes with the focal root (*v* or *u*). More detail of this merging operation is given below at the "Tree editing operations and their costs" section below. The node pairs and editing operations (pruning and/or merging) giving rise to the optimal alignment was stored for later traceback.

These processes described above went recursively until the whole DP matrix was filled. For the global alignment between *Q* and *S*, the traceback procedure starts from *w*(*Q*,*S*), i.e., the one DP matrix cell representing the alignment between the roots of *Q* and that of *S*. The optimal alignment for each node was then determined by the optimal choice recorded in each DP matrix cell along the (branched) route of recursive traceback. For the local alignment, the one DP matrix cell with the highest *w* (not necessarily involving the root of CLT) was located, and the local alignment was extracted by recursive traceback starting from this very DP matrix cell. All the DP matrix cells on the route of back tracing were marked as used. To extract the second local alignment, the highest *w* in the unused DP matrix cells whose entire back-trace with at least 90% of unused matrix cells will be used. The 90% unused matrix cell criteria prevent multiple high score alignments created by the same pair of homeomorphic subtrees with minor additions or modifications. In a similar manner, more local alignments can be determined from the DP matrix.

Cell-to-cell alignment scores

In the first stage of the dynamic programming procedure in mDELTA, each pair of terminal

cells consisting one from tree Q and one from tree S is scored by their transcriptomic similarity. Compared to our previous DELTA implementation, which used a user-defined matrix listing scores for all pairs of cell types, the single-cell transcriptome-based score provides a more subjective assessment of cell similarity. More specifically, the expression distance between two single cells, denoted as d, is calculated as the Euclidean distance of their transcriptomes (expression levels of all genes), which is then transformed to a similarity score as $s = -log(d+1)$. The similarity scores for all cell pairs were further scaled to s', so that the 45% most similar cell pairs have $s' > 0$, meaning that they are positive contributors to the alignment, and the 15% least similar cell pairs have $s' < -1$. In the case of a cell pair with $s' < -1$ being chosen by the Hungarian algorithm as part of the optimal solution, both cells within this pair will be removed from the alignment and the alignment score of the optimal solution will be subtracted by 1. This means that the pair of cells is so different that removing them is better than aligning them (a severely penalized mismatch). Note that this only happen to pairs of cells but not pairs of internal nodes, so it is different from the pruning of a subtree (see the "Tree editing operations and their costs" below). Finally, the alignment score between a pair of cell types x and y is defined as the average similarity score between all pairs of cells containing one *x* cell and one *y* cell.

Tree editing operations and their costs

First, changing the order of the daughters of an internal node, along with their descendant subtrees, is not penalized by mDELTA, as genomic-barcoding-based CLTs are unordered trees.

Second, mDELTA finds alignments between two trees with the necessary "pruning" of some subtrees. The pruning operation is required because some cells (and therefore their corresponding sub-CLTs) are not captured by the 10x Chromium single-cell transcriptome library preparation pipeline (top panel in the schematic diagram below). For example, consider two identical actual CLTs Q and S and their experimentally-reconstructed CLT $Q'$and S', where a sub-CLT in Q is missed in Q' due to the above problem. By pruning the corresponding sub-CLT in S', the correct cell-to-cell alignment between Q and S may be retrieved in the isomorphic alignment between $Q'$and S'. A pruning of subtree T_v^u includes three steps. (i) Remove the edge $(u \to v)$ and all the nodes and edges in T_v^u; (ii) If *u* has only two remaining neighbors attached to it by $(u' \to u)$ and $(u \to u'')$, connect them by a new edge $(u' \to u'')$; (iii) Remove *u* and all edges attached to it. In our implementation, pruning is penalized by a pruning cost *p*, such that $p(\hat{T})$ as in formula [1] equals to *p* times the number of terminal nodes in $\hat{T}$, the pruned subtree. We used *p*=0.2 across our study. An increased cost may enhance alignment specificity and decreased cost may enhance sensitivity. The choice of *p*=0.2 along with the cell-to-cell alignment score described above represents a compromise between specificity and sensitivity. Additionally, any alignment with >20% node pruned in either subtree is not reported by mDELTA.

Third, a “merging” operation is considered by mDELTA to facilitate alignment. The merging operation reflects the unresolved cell division events due to intermittent barcode editing (bottom panel in the schematic diagram below). For example, consider two identical actual CLTs *Q* and *S* and their experimentally-reconstructed CLT *Q'* and *S'*, where no barcode editing happened in the cell cycle of an internal cell *v* in Q, making one bifurcating internal node *u* (the mother cell of *v*) appear as trifurcating in *Q'* (i.e. *u* is directly connected to its other daughter *u'*, and the two daughters of *v*). By merging the node corresponding to *v* with that corresponding to *u* in *S'*, the correct cell-to-cell alignment between *Q* and *S* may be retrieved in the isomorphic alignment between *Q'* and *S'*. A merging of an internal node *v* to its mother node (i.e., they are connected by $(u \rightarrow v)$) includes two steps. (i) For all out-going edges from *v* (i.e. $(v \rightarrow v')$, $(v \rightarrow v'')$, ...), change their origin to *u* (i.e. $(u \rightarrow v')$, $(u \rightarrow v'')$, ...). (ii) Remove *v* and all edges attached to it. Merging is penalized by a merging coefficient *m*, such that $m(\hat{v})$ in formula [1] equals to *m*. In other words, the total cost of merging for an alignment equals *m* times the number of internal nodes being merged. We used *m*=100 in our study in order to avoid "over-aligned" results caused by excessive flexibility introduced by merging too many internal nodes with the root.

A schematic diagram showing examples of necessary pruning and merging is given below.

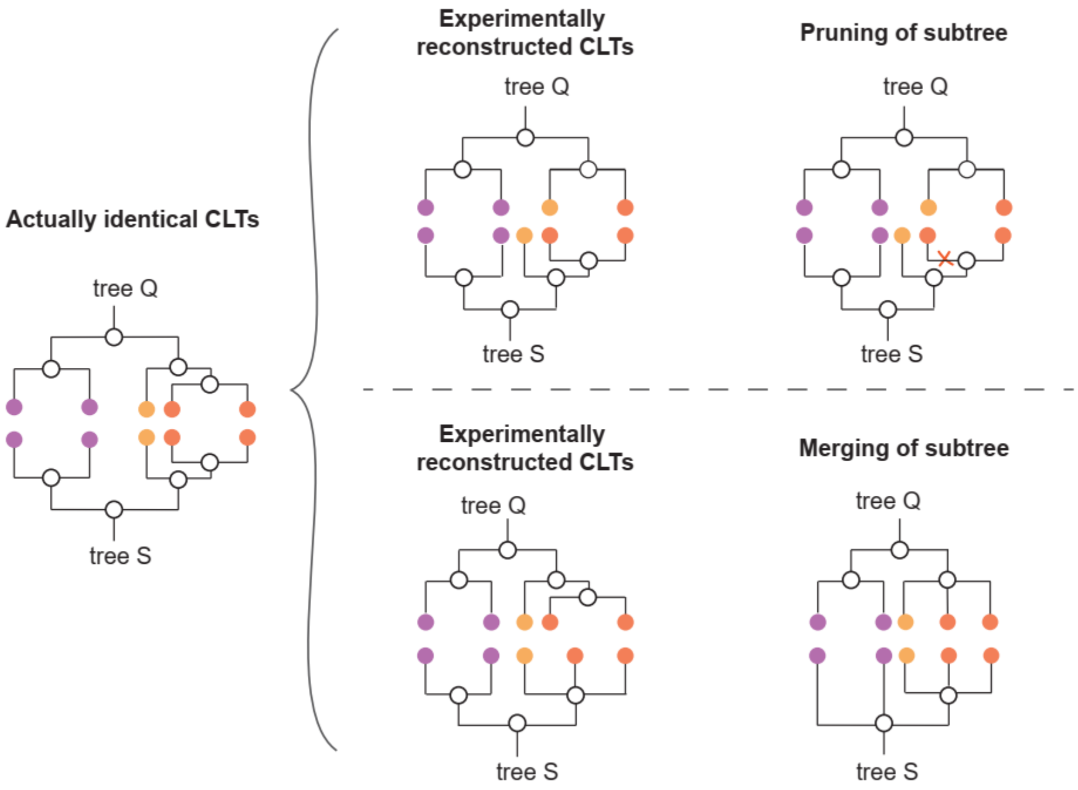

Video S1. Alveolospheres developed on day 15 of the in vitro directed differentiation

(video in a separated file)

Following the sorting and replating of NKX2-1^{+} lung progenitors on day 15, alveolospheres are developed in 3D Matrigel culture with CK+DCI media within 3-7 days and maintained in CK+DCI media for weeks. These spheres are examined by Z stack live images on the Leica DMi8 fluorescence microscope.

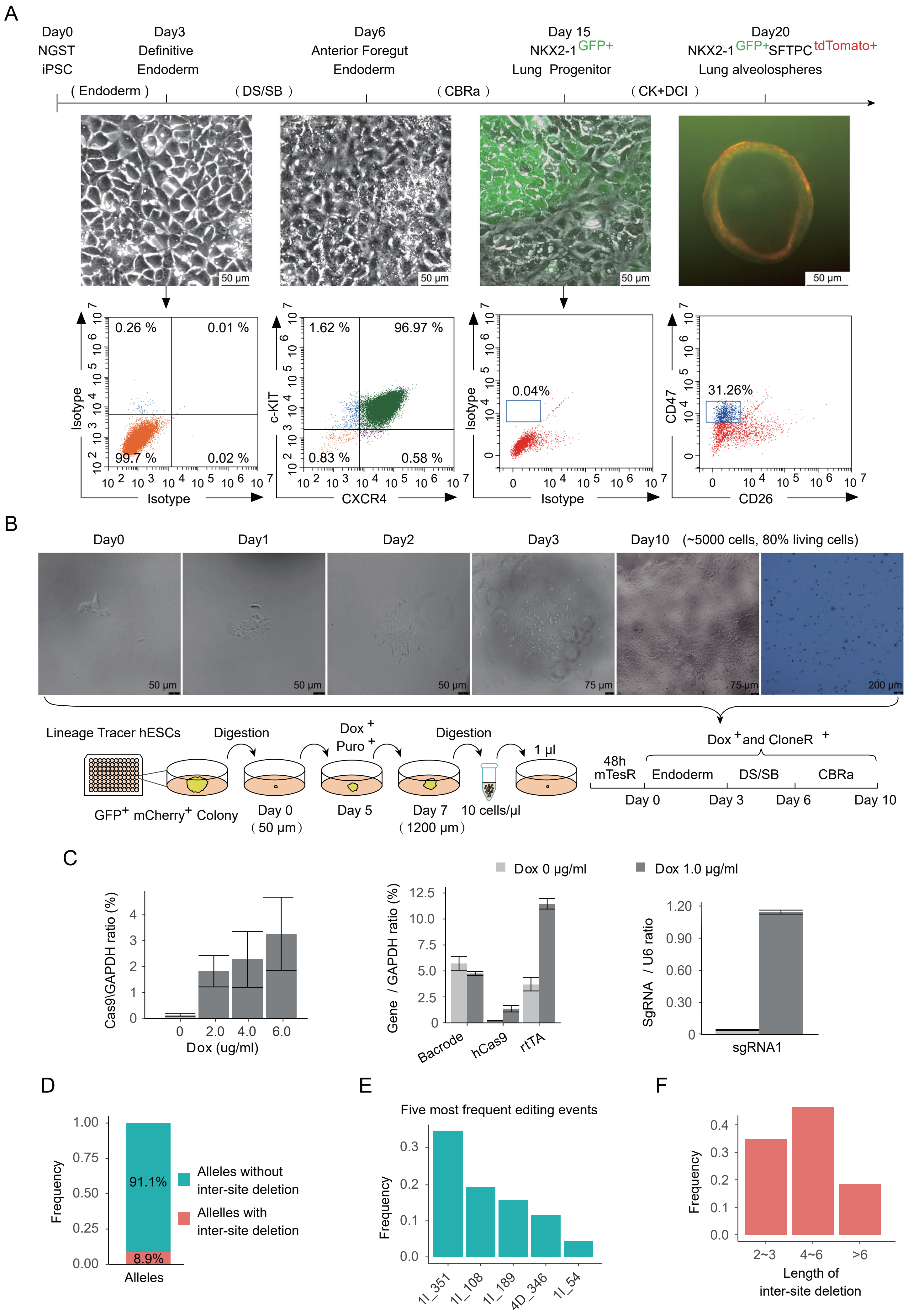
A
Day0 NGST iPSC
Day3 Definitive Endoderm
Day6 Anterior Foregut Endoderm
Day 15 NKX2-1 GFP+ Lung Progenitor
Day20 NKX2-1 GFP+ SFTPC tdTomato+ Lung alveolospheres
(Endoderm)
(DS/SB)
(CBRa)
(CK+DCI)
50 μm
0.26 %
0.01 %
99.7 %
0.02 %
1.62 %
96.97 %
0.83 %
0.58 %
0.04%
31.26%
Isotype
c-KIT
CXCR4
CD47
CD26
B
Day0
Day1
Day2
Day3
Day10 (~5000 cells, 80% living cells)
75 μm
200 μm
Lineage Tracer hESCs
Digestion
Dox +
Puro +
Digestion
1 μl
GFP+ mCherry+ Colony
Day 0 (50 μm)
Day 5
Day 7 (1200 μm)
10 cells/μl
48h mTesR
Dox + and CloneR +
Endoderm
DS/SB
CBRa
Day 0
Day 3
Day 6
Day 10
C
Cas9\GAPDH ratio (%)
Dox (ug/ml)
Dox 0 μg/ml
Dox 1.0 μg/ml
Gene / GAPDH ratio (%)
Bacrode
hCas9
rtTA
SgRNA / U6 ratio
sgRNA1
D
Frequency
91.1%
8.9%
Alleles
Alleles without inter-site deletion
Alleles with inter-site deletion
E
Five most frequent editing events
Frequency
1I_351
1I_108
1I_189
4D_346
1I_54
F
Frequency
2~3
4~6
>6
Length of inter-site deletion

Figure S1. Reliability of the directed differentiation and experimental lineage tracing

(**A**) Verification of in vitro directed differentiation toward PLP at hallmark steps ranging from day 0 (hESC), day 3 (definitive endoderm, with flow cytometry results below), day 6 (anterior foregut endoderm), day 15 (primordial lung progenitor, with flow cytometry results below) to day 20 (Lung alveolar type II epithelial cells, fluorescence imaging) by using the BU3 NGST (NKX2-1-GFP; SFTPC-tdTomato) iPS cell line. Bars at the bottom right corners indicate 50 μm. **(B)** Key steps of experimental lineage tracing for in vitro directed differentiation from several (~10) lineage tracer hESCs (sgRNA-mCherry; lineage barcode-GFP) to PLP are shown at the bottom. The process began with the selection of traceable colonies (GFP^+ and $mCherry^+$) by a 7-day culture, during which a brief Cas9 induction was applied to uniquely label the ancestor cells by the resulting mutations on the lineage barcode. The selected colonies were digested and plated again at ~10 cells per well for the subsequent directed differentiation culture, which lasted for 10 days to produce ~5000 cells. On top, a typical sample is shown with bright field images at several timepoints, with the scale bar placed at the bottom right corner. **(C)** Cas9 (left), the lineage barcode (middle), and sgRNAs (right) are sufficiently expressed/induced in lineage tracer hESCs. The error bars indicate the standard error of three replicates. **(D)** The frequency of inter-site (red) and non-inter-site (blue) deletions found in edited barcode of lineage tracer hESCs. **(E)** The most frequent editing events are evenly dispersed within the lineage barcode. Editing events are named (*x* axis) by length (the number before I/D), type (I: insertion; D: deletion) and position (the number after the underline). **(F)** The frequency of inter-site deletion events of different lengths (in terms of the number of editing sites) among all inter-site deletion events.

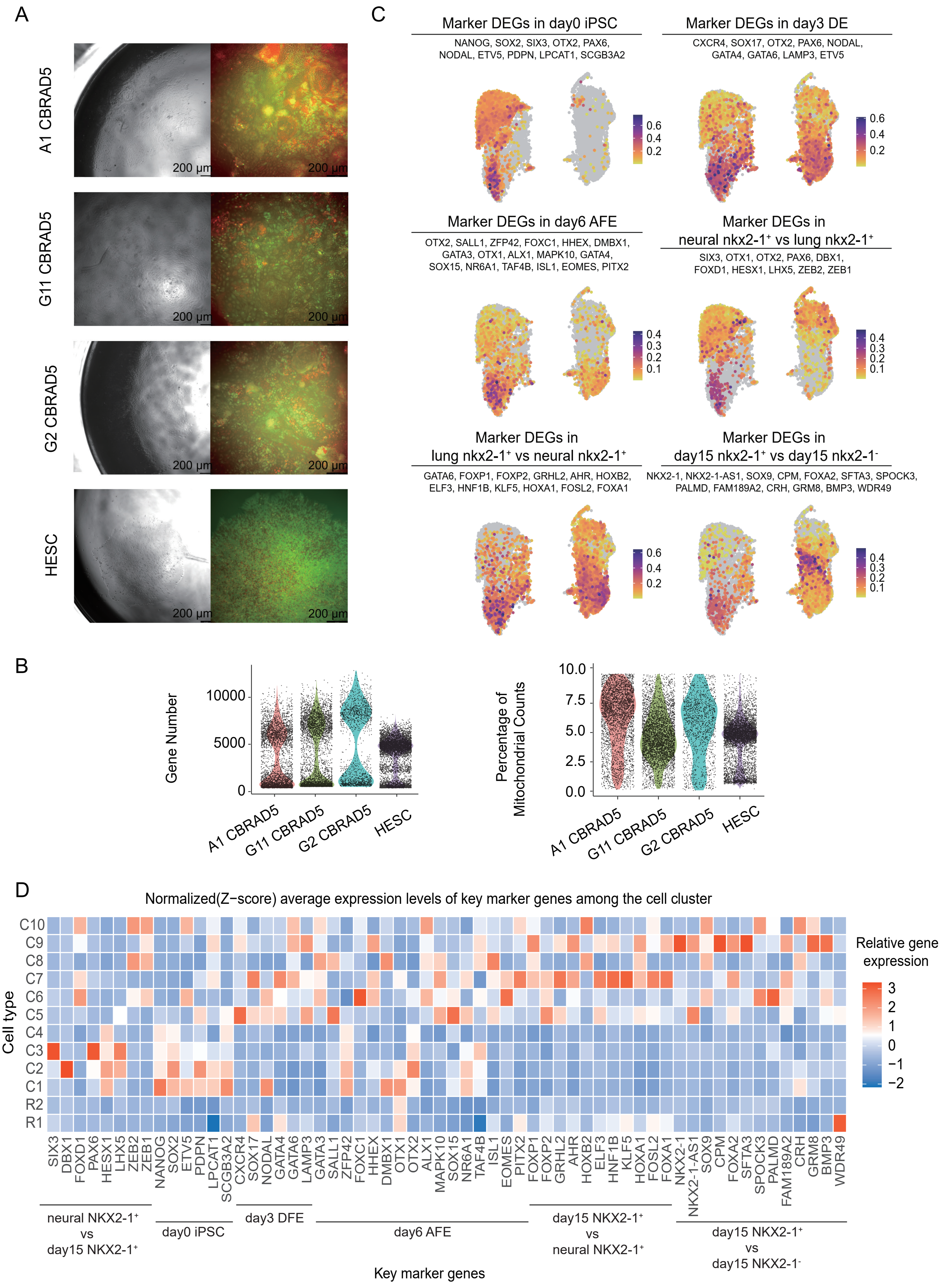
A
A1 CBRAD5
G11 CBRAD5
G2 CBRAD5
HESC
200 μm
C
Marker DEGs in day0 iPSC
NANOG, SOX2, SIX3, OTX2, PAX6, NODAL, ETV5, PDPN, LPCAT1, SCGB3A2
Marker DEGs in day3 DE
CXCR4, SOX17, OTX2, PAX6, NODAL, GATA4, GATA6, LAMP3, ETV5
Marker DEGs in day6 AFE
OTX2, SALL1, ZFP42, FOXC1, HHEX, DMBX1, GATA3, OTX1, ALX1, MAPK10, GATA4, SOX15, NR6A1, TAF4B, ISL1, EOMES, PITX2
Marker DEGs in neural nkx2-1⁺ vs lung nkx2-1⁺
SIX3, OTX1, OTX2, PAX6, DBX1, FOXD1, HESX1, LHX5, ZEB2, ZEB1
Marker DEGs in lung nkx2-1⁺ vs neural nkx2-1⁺
GATA6, FOXP1, FOXP2, GRHL2, AHR, HOXB2, ELF3, HNF1B, KLF5, HOXA1, FOSL2, FOXA1
Marker DEGs in day15 nkx2-1⁺ vs day15 nkx2-1⁻
NKX2-1, NKX2-1-AS1, SOX9, CPM, FOXA2, SFTA3, SPOCK3, PALMD, FAM189A2, CRH, GRM8, BMP3, WDR49
B
Gene Number
10000
5000
0
A1 CBRAD5
G11 CBRAD5
G2 CBRAD5
HESC
Percentage of Mitochondrial Counts
10.0
7.5
5.0
2.5
0.0
D
Normalized(Z−score) average expression levels of key marker genes among the cell cluster
Cell type
C10
C9
C8
C7
C6
C5
C4
C3
C2
C1
R2
R1
Relative gene expression
SIX3 DBX1 FOXD1 PAX6 HESX1 LHX5 ZEB2 ZEB1 NANOG SOX2 ETV5 PDPN LPCAT1 SCGB3A2 CXCR4 SOX17 NODAL GATA4 GATA6 LAMP3 GATA3 SALL1 ZFP42 FOXC1 HHEX DMBX1 OTX1 OTX2 ALX1 MAPK10 SOX15 NR6A1 TAF4B ISL1 EOMES PITX2 FOXP1 FOXP2 GRHL2 AHR HOXB2 ELF3 HNF1B KLF5 HOXA1 FOSL2 FOXA1 NKX2-1 NKX2-1-AS1 SOX9 CPM FOXA2 SFTA3 SPOCK3 PALMD FAM189A2 CRH GRM8 BMP3 WDR49
neural NKX2-1⁺ vs day15 NKX2-1⁺
day0 iPSC
day3 DFE
day6 AFE
day15 NKX2-1⁺ vs neural NKX2-1⁺
day15 NKX2-1⁺ vs day15 NKX2-1⁻
Key marker genes

Figure S2. Quality of the directed differentiation

(A) Morphology and fluorescence imaging of differentiating/CBRAD5 and hESC self-renewal samples on day 10. **(B)** Overview of single-cell transcriptomes measured for differentiating and hESC samples. The slightly increased percentage of mitochrondial reads is not a sign of Dox toxicity, but rather a known feature of differentiation[2-6]. **(C)** Feature plots for average expression level of marker DEGs found in previous microarray-based transcriptomes of specific developmental stages[7], based on UMAP visualization of single-cell transcriptomes as described in **Figure 1B**. The specific marker genes were listed below the title and above the plot. (**D**) Normalized (Z-score) average expression levels (color scale on top) of key marker genes (*x* axis) in the cell clusters (*y* axis) were shown as a heatmap. There are labelled horizontal lines below the gene names that indicate the developmental stages associated with each marker gene.

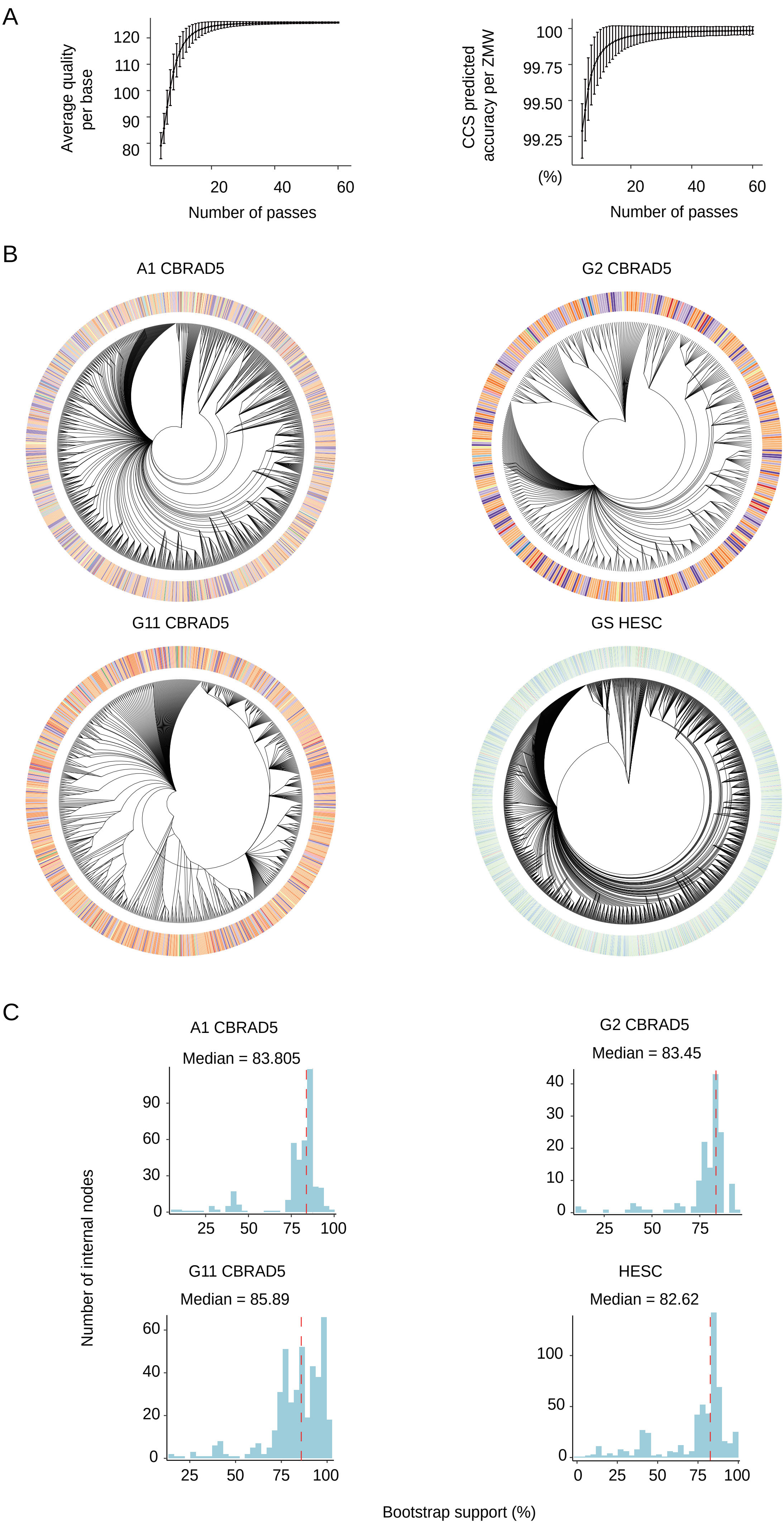
A
Average quality per base
120
110
100
90
80
20
40
60
Number of passes
CCS predicted accuracy per ZMW
100
99.75
99.50
99.25
(%)
20
40
60
Number of passes
B
A1 CBRAD5
G2 CBRAD5
G11 CBRAD5
GS HESC
C
A1 CBRAD5
Median = 83.805
G2 CBRAD5
Median = 83.45
G11 CBRAD5
Median = 85.89
HESC
Median = 82.62
Number of internal nodes
Bootstrap support (%)

Figure S3. Quality of the lineage tracing

(A) Sequencing quality and accuracy of PacBio HiFi-reads given the required number of passes. Error bars indicates standard deviation among ZMWs. **(B)** Tree representation of the CLTs shown in **Figure 1G**. **(C)** Bootstrap support percentages for the internal nodes of the CLTs in each sample are presented as histograms. The sample names and median bootstrap support are shown in the plot titles and in-plot texts, respectively, with the median support further indicated by a red vertical dashed line.

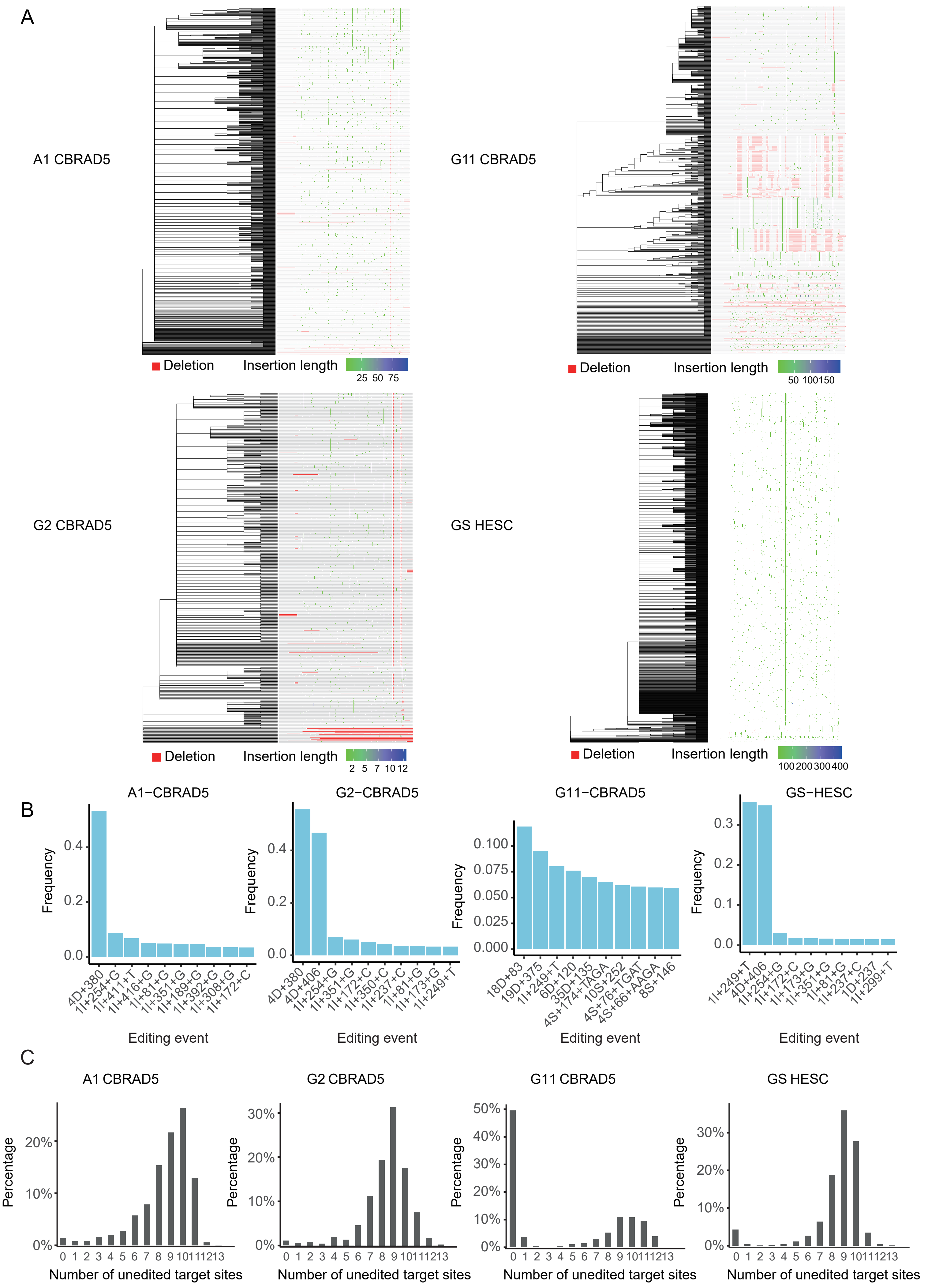
A
A1 CBRAD5
Deletion
Insertion length
25 50 75
G11 CBRAD5
Deletion
Insertion length
50 100150
G2 CBRAD5
Deletion
Insertion length
2 5 7 10 12
GS HESC
Deletion
Insertion length
100 200 300 400
B
A1−CBRAD5
Frequency
Editing event
4D+380
1I+254+G
1I+411+T
1I+416+G
1I+81+G
1I+351+G
1I+189+G
1I+392+G
1I+308+G
1I+172+C
G2−CBRAD5
4D+380
4D+406
1I+254+G
1I+351+G
1I+172+C
1I+350+C
1I+237+C
1I+81+G
1I+173+G
1I+249+T
G11−CBRAD5
18D+83
19D+375
1I+249+T
6D+120
35D+135
4S+174+TAGA
10S+252
4S+76+TGAT
4S+66+AAGA
8S+146
GS−HESC
1I+249+T
4D+406
1I+254+G
1I+172+C
1I+173+G
1I+351+G
1I+81+G
1I+237+C
1D+237
1I+299+T
C
A1 CBRAD5
G2 CBRAD5
G11 CBRAD5
GS HESC
Percentage
Number of unedited target sites

Figure S4. More details on the editing status of the lineage barcode

(A) Editing status of the lineage barcode in each terminal node of the lineage trees. **(B)** The prevalence (in terms of fraction of tips harboring it) of the top editing events in each sample. The editing events were encoded as a string with three parts separated by "+", with the first part being the CIGAR string of the specific type of edit, the second part being the position within the lineage barcode where the editing occurs, and the third part being the inserted sequence (if any). **(C)** The number of intact editable sites within each unique barcode were shown as a histogram for each sample.

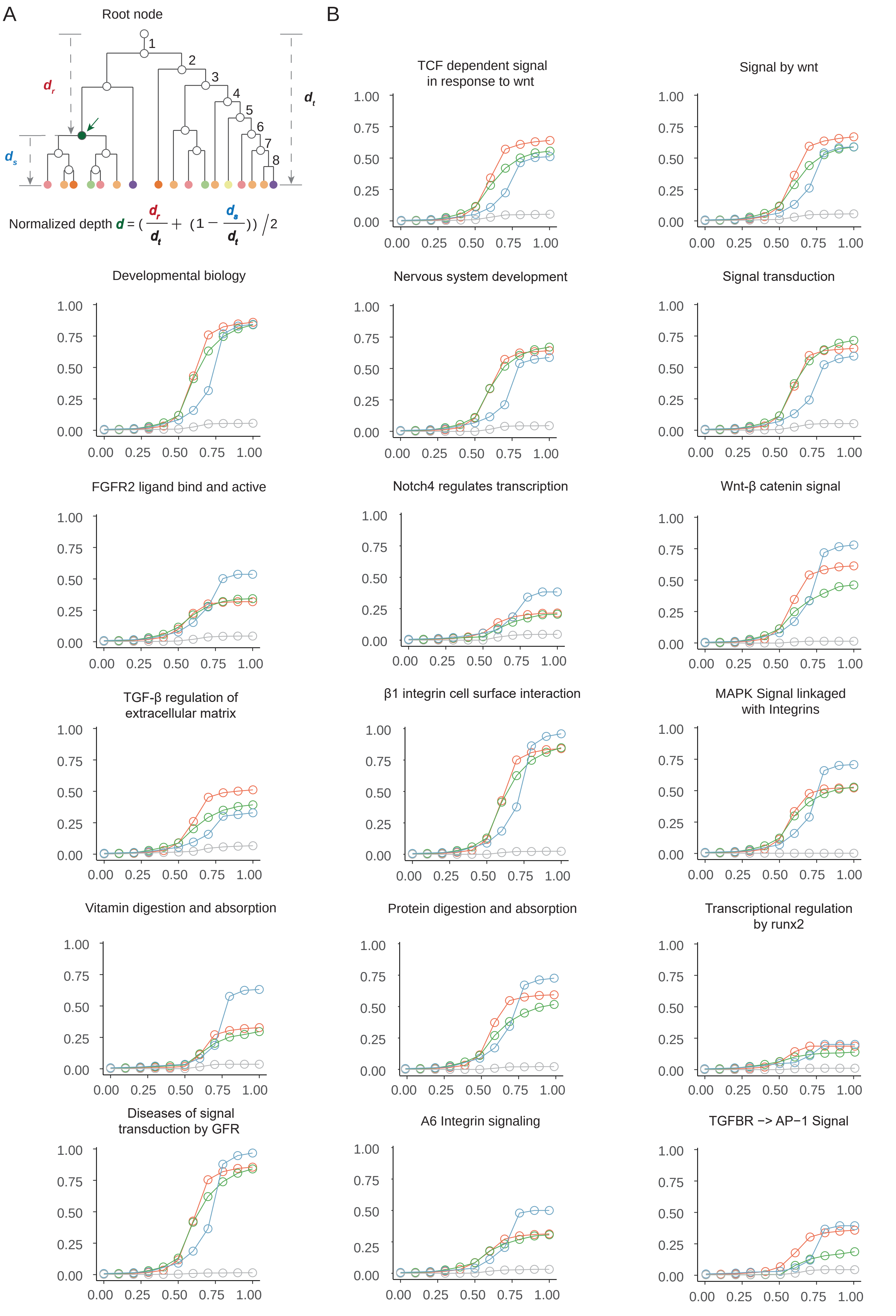
A
Root node
1
2
3
4
5
6
7
8
d_r
d_s
d_t
Normalized depth $d = (\frac{d_r}{d_t} + (1 - \frac{d_s}{d_t}))/2$
B
TCF dependent signal in response to wnt
Signal by wnt
Developmental biology
Nervous system development
Signal transduction
FGFR2 ligand bind and active
Notch4 regulates transcription
Wnt-β catenin signal
TGF-β regulation of extracellular matrix
β1 integrin cell surface interaction
MAPK Signal linkaged with Integrins
Vitamin digestion and absorption
Protein digestion and absorption
Transcriptional regulation by runx2
Diseases of signal transduction by GFR
A6 Integrin signaling
TGFBR –> AP−1 Signal
0.00
0.25
0.50
0.75
1.00

Figure S5. Transcriptional divergence among sub-CLTs

(**A**) Schematic diagram for the normalized depth of a node (see **Methods**). (**B**) Same as **Figure 2F** except that the analyses were limited to specific GO terms indicated on top of each panel.

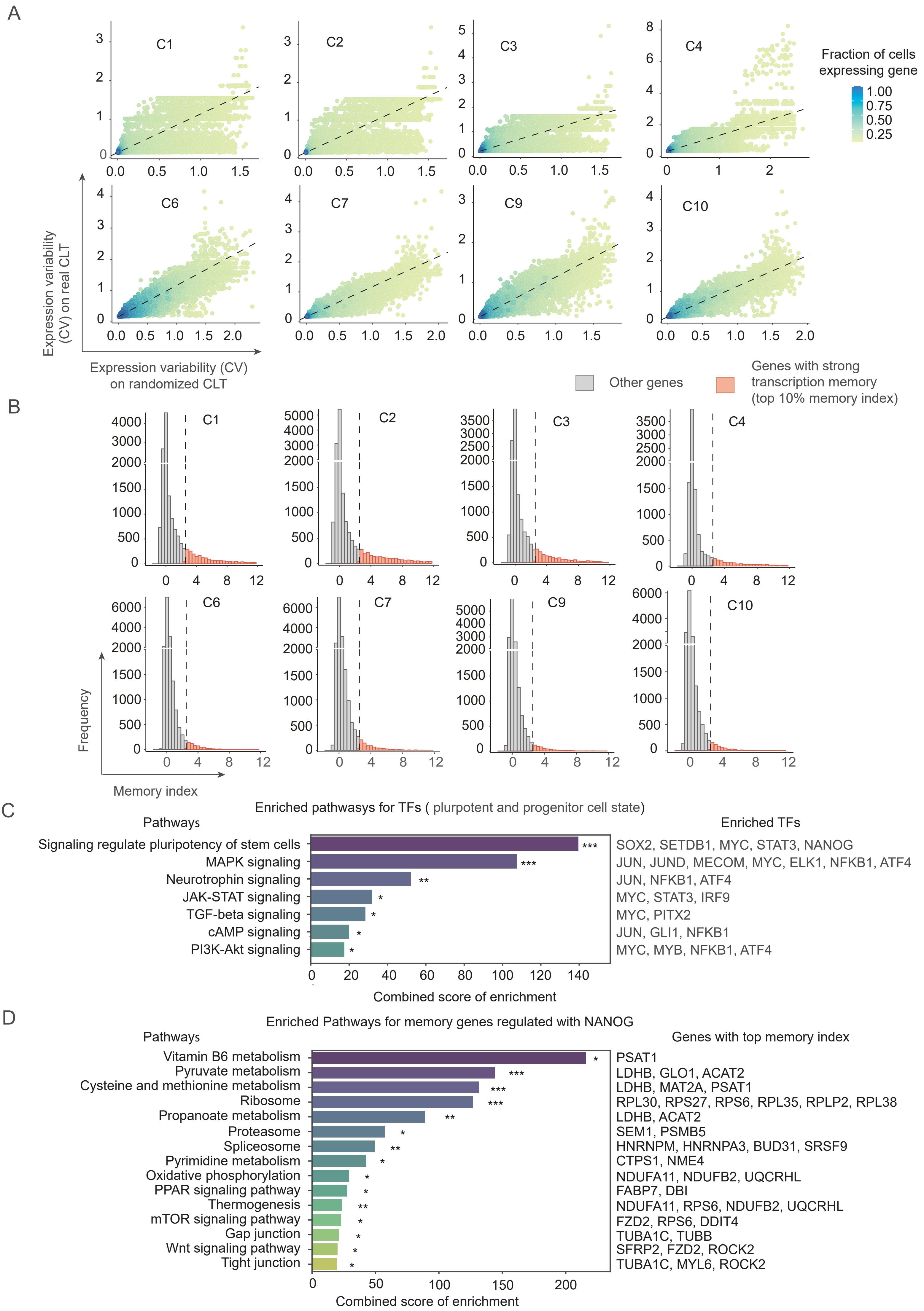
A
C1
C2
C3
C4
C6
C7
C9
C10
Fraction of cells expressing gene
1.00
0.75
0.50
0.25
Expression variability (CV) on real CLT
Expression variability (CV) on randomized CLT
B
Other genes
Genes with strong transcription memory (top 10% memory index)
Frequency
Memory index
C
Enriched pathwasys for TFs (plurpotent and progenitor cell state)
Pathways
Enriched TFs
Signaling regulate pluripotency of stem cells
MAPK signaling
Neurotrophin signaling
JAK-STAT signaling
TGF-beta signaling
cAMP signaling
PI3K-Akt signaling
SOX2, SETDB1, MYC, STAT3, NANOG
JUN, JUND, MECOM, MYC, ELK1, NFKB1, ATF4
JUN, NFKB1, ATF4
MYC, STAT3, IRF9
MYC, PITX2
JUN, GLI1, NFKB1
MYC, MYB, NFKB1, ATF4
Combined score of enrichment
D
Enriched Pathways for memory genes regulated with NANOG
Pathways
Genes with top memory index
Vitamin B6 metabolism
Pyruvate metabolism
Cysteine and methionine metabolism
Ribosome
Propanoate metabolism
Proteasome
Spliceosome
Pyrimidine metabolism
Oxidative phosphorylation
PPAR signaling pathway
Thermogenesis
mTOR signaling pathway
Gap junction
Wnt signaling pathway
Tight junction
PSAT1
LDHB, GLO1, ACAT2
LDHB, MAT2A, PSAT1
RPL30, RPS27, RPS6, RPL35, RPLP2, RPL38
LDHB, ACAT2
SEM1, PSMB5
HNRNPM, HNRNPA3, BUD31, SRSF9
CTPS1, NME4
NDUFA11, NDUFB2, UQCRHL
FABP7, DBI
NDUFA11, RPS6, NDUFB2, UQCRHL
FZD2, RPS6, DDIT4
TUBA1C, TUBB
SFRP2, FZD2, ROCK2
TUBA1C, MYL6, ROCK2
Combined score of enrichment

Figure S6. Transcriptional memory in individual cell types

(**A** and **B**) Similar to **Figure 3B** and **C**, except that each major cell type was plotted separately.

(**C**) The transcription factors appeared in **Figure 3E** are collectively tested for enrichment in GO terms by Enrichr. Combined score of enrichment is calculated by Enrichr (see **Methods**). The statistical significance of enrichment according to Fisher's exact test is indicated as *:P<0.05; **:P<0.01; ***:P<0.001.

(**D**) Similar to **C**, except Genes with top memory indices and regulated by NANOG are tested for enrichment in GO terms by Enrichr.

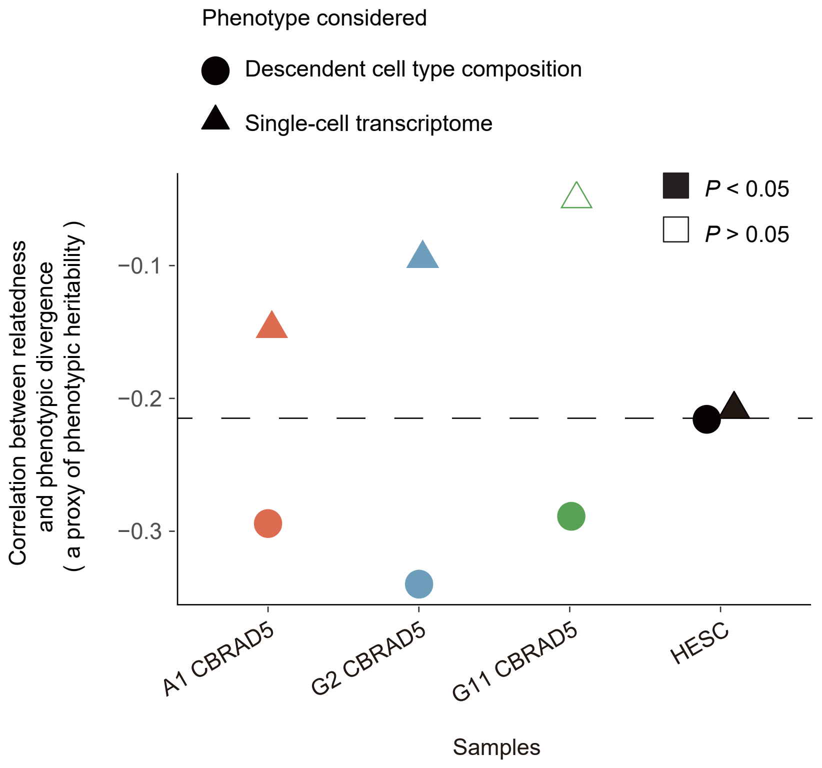

Phenotype considered
Descendent cell type composition
Single-cell transcriptome
P < 0.05
P > 0.05
Correlation between relatedness and phenotypic divergence (a proxy of phenotypic heritability)
−0.1
−0.2
−0.3
A1 CBRAD5
G2 CBRAD5
G11 CBRAD5
HESC
Samples

Figure S7. Heritability of descendent cell type compositions and single-cell transcriptomes

A proxy of the phenotypic heritability, the Spearman's Correlation Coefficient (*y* axis) between relatedness and phenotypic divergence, is calculated for all pairs of relevant nodes (see **Methods**). For the differentiating samples (*x* axis), the correlation and therefore phenotypic heritability is always stronger for the phenotype of descendent cell type components (dots) compared to single-cell transcriptomes (triangles). The correlation is nevertheless indistinguishable between the two phenotypes in the non-differentiating sample. A filled or empty point is used to indicate whether the correlation is statistically significant. A slight offset has been applied to the points of the two phenotypes in order to avoid overplotting.

References

1 Yuan, M. *et al.* Alignment of Cell Lineage Trees Elucidates Genetic Programs for the Development and Evolution of Cell Types. *iScience* **23**, 101273 (2020). https://doi.org:10.1016/j.isci.2020.101273

2 Peaslee, C. *et al.* Doxycycline Significantly Enhances Induction of Induced Pluripotent Stem Cells to Endoderm by Enhancing Survival Through Protein Kinase B Phosphorylation. *Hepatology* **74**, 2102-2117 (2021). https://doi.org:10.1002/hep.31898

3 Zamai, L. *et al.* In vitro apoptotic cell death during erythroid differentiation. *Apoptosis* **9**, 235-246 (2004). https://doi.org:10.1023/B:APPT.0000018805.63663.a5

4 Levine, A. J. The paths to death and differentiation. *Cell Death & Differentiation* **18**, 1391-1392 (2011). https://doi.org:10.1038/cdd.2011.41

5 Malin, J. Z. & Shaham, S. Cell Death in C. elegans Development. *Curr Top Dev Biol* **114**, 1-42 (2015). https://doi.org:10.1016/bs.ctdb.2015.07.018

6 Osorio, D. & Cai, J. J. Systematic determination of the mitochondrial proportion in human and mice tissues for single-cell RNA-sequencing data quality control. *Bioinformatics* **37**, 963-967 (2021). https://doi.org:10.1093/bioinformatics/btaa751

7 Hawkins, F. *et al.* Prospective isolation of NKX2-1-expressing human lung progenitors derived from pluripotent stem cells. *J Clin Invest* **127**, 2277-2294 (2017). https:// doi: 10.1172/JCI89950
